# Direct Detection of *Streptococcus suis* from Cerebrospinal Fluid, Positive Hemoculture, and Simultaneous Differentiation of Serotypes 1, 1/2, 2, and 14 within Single Reaction

**DOI:** 10.3390/pathogens10080996

**Published:** 2021-08-06

**Authors:** Ingyin Shun Lae Thu, Khajornsak Tragoolpua, Sorasak Intorasoot, Usanee Anukool, Phadungkiat Khamnoi, Anusak Kerdsin, Chayada Sitthidet Tharinjaroen

**Affiliations:** 1 Division of Clinical Microbiology, Faculty of Associated Medical Sciences, Chiang Mai University, Chiang Mai 50200, Thailand; ingyinshun_laethu@cmu.ac.th (I.S.L.T.); khajornsak.tr@cmu.ac.th (K.T.); sorasak.in@cmu.ac.th (S.I.); usanee.anukool@cmu.ac.th (U.A.); 2 Infectious Diseases Research Unit (IDRU), Faculty of Associated Medical Sciences, Chiang Mai University, Chiang Mai 50200, Thailand; 3 Microbiology Unit, Diagnostic Laboratory, Maharaj Nakorn Chiang Mai Hospital, Chiang Mai 50200, Thailand; micromedcmu@hotmail.com; 4 Faculty of Public Health, Kasetsart University, Chalermphrakiat Sakon Nakhon Province Campus, Sakon Nakhon 47000, Thailand; noksak99@gmail.com

**Keywords:** *Streptococcus suis*, multiplex PCR, direct detection, serotyping, serotype 1, serotype 1/2, serotype 2, serotype 14

## Abstract

*Streptococcus suis* is an emerging zoonotic bacterium causing septicemia and meningitis in humans. Due to rapid disease progression, high mortality rate, and many underdiagnosed cases by time-consuming routine identification methods, alternative diagnostic testing is essential. Among 29 broadly accepted *S. suis* serotypes, serotypes 2 and 14 are high prevalent; however, many PCR assays showed an inability to differentiate serotype 2 from 1/2, and 1 from 14. In this study, we developed and validated a new multiplex PCR assay that facilitates the identification of only the 29 true serotypes of *S. suis* and simultaneously differentiates serotypes 1, 1/2, 2, and 14 within a single reaction. Importantly, the multiplex PCR could detect *S. suis* directly from positive hemocultures and CSF. The results revealed high sensitivity, specificity, and 100% accuracy with almost perfect agreement (κ = 1.0) compared to culture and serotyping methods. Direct detection enables a decrease in overall diagnosis time, rapid and efficient treatment, reduced fatality rates, and proficient disease control. This multiplex PCR offers a rapid, easy, and cost-effective method that can be applied in a routine laboratory. Furthermore, it is promising for developing point-of-care testing (POCT) for *S. suis* detection in the future.

## 1. Introduction

*Streptococcus suis*, a Gram-positive cocci bacterium, is one of the leading causative agents of massive economic losses in the pig industry. It is also an emerging zoonotic infectious organism that has been receiving growing concern around the world [1,2]. *S. suis* naturally colonizes in the upper respiratory tract of pigs, particularly in tonsils and nasal cavities. On the other hand, it is responsible for meningitis, arthritis, pericarditis, polyserositis, septicemia, and sudden death of weaning piglets, as well as growing pigs [3]. Humans can be infected when in close contact with diseased pigs or consuming *S. suis* contaminated raw pork or pork-derived products [1]. Various symptoms, including fever, headache, septicemia, deafness to severe septic shock syndrome, and fatality, are found. The number of reported human cases has been dramatically increased in the last decade, and the vast majority are from Asia (90%) [4]. To date, *S. suis* has been re-classified into 29 serotypes based on genetic analysis [5,6]. Previously, *S. suis* has been recognized as 35 serotypes (serotype 1/2, and 1–34); however, serotypes 20, 22, 26, 32, 33, and 34 were re-identified into other species and are referred to as *S. suis*-like bacterium [7,8,9,10,11]. As not all serotypes are related to zoonotic potential and disease severity, it is important to know information about serotypes. Only some serotypes, such as serotypes 2 and 14, are associated with zoonotic infection; whereas, serotype 2 is the most prevalent serotype in both humans and pigs worldwide [12]. However, *S. suis* serotypes 1, 4, 5, 9, 16, 21, 24, and 31 have also been isolated from patients [13,14,15,16,17,18,19,20,21]. Serotype 1/2 is the most predominant serotype found in diseased pigs in North America [22]. In Thailand, the annual incidence rate of 0.51/100,000 persons has been reported by the Bureau of Epidemiology, Ministry of Public Health in 2020 (http://www.boe.moph.go.th/boedb/surdata/disease.php?dcontent=situation&ds=82, accessed on 15 July 2021). Serotype 2 is the most common serotype found in human *S. suis* infection cases (93.4%). The second most common serotype is serotype 14 (5.2%) followed by some others such as 24 (0.6%), 5 (0.4%), 4 (0.1%), 9 (0.1%), and 31 (0.1%) [15,16,17,23,24,25,26]. Serotyping is a vital diagnostic method for understanding the epidemiology of an outbreak, monitoring the prevalence of specific *S. suis* serotypes, and guiding vaccine development.

Serological typing was traditionally performed by a coagglutination test, precipitation test, and Neufeld’s capsular reaction test using reference antisera which is available for all serotypes in only a few laboratories around the world [27,28]. So far, many PCR-based serotypes have been developed based on capsular polysaccharide encoding gene (*cps*) [5,29,30,31]. Unfortunately, several PCR-based detection methods are unable to differentiate serotype 2 from 1/2, and serotype 1 from 14 as the *cps* gene of these serotypes are highly homologous [5]. However, it has been reported that a single nucleotide polymorphism (SNP) in the *cpsK* gene of these serotypes, at position 483, could differentiate between serotypes 2 and 1/2, as well as 1 and 14 [32]. The serotype 2 and 14 strains possess G nucleotide in contrast to serotype 1 and 1/2, which possess C or T at position 483 of the *cpsK* gene. PCR derivatives such as real-time PCR with a high resolution melting curve analysis, PCR-restriction fragment length polymorphism (PCR-RFLP), and mismatch amplification mutation assay (MAMA-PCR) have been applied to differentiate among serotypes 2, 1/2, 1, and 14 [33,34,35]. However, these PCR assays are sophisticated and require multiple steps or special instruments.

In this study, we reported a simple, cheap, high accuracy multiplex PCR assay for differentiating among serotypes 1, 1/2, 2, and 14 based on the targeted *cpsJ* gene, the SNP in *cpsK* gene, and also the identification of *S. suis* using DNA repair protein gene (*recN* gene) that can differentiate *S. suis* from *S. suis*-like strains. This new multiplex PCR assay was validated using human and pig isolates. Moreover, we present the ability of the multiplex PCR to direct the detection of *S. suis* and serotypes 1, 1/2, 2, and 14 from clinical specimens by omitting the culture step. The developed assay will provide an alternative simple, rapid, high accuracy diagnostic test that allows access to well-organized treatment to reduce the severity and mortality rate of the infected patient. 

## 2. Results

### 2.1. New Designed Primers and Optimization of Multiplex PCR 

**Figure 1:**
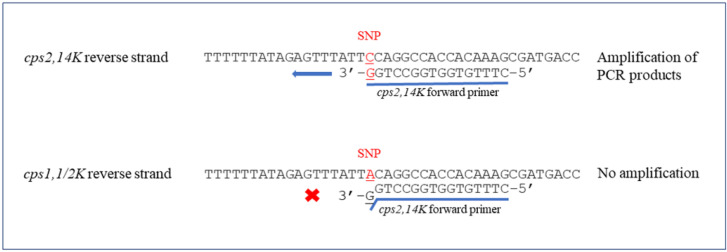
Schematic representation of *cps2,14K* forward primer binding site. The SNP at position 483 of *cpsK* gene were indicated in red. **×** represented no amplification and ← showed the ability to amplify PCR products.

Two new pairs of primers were designed in this study; one pair (species-specific) primer was based on the *recN* gene to identify *S. suis* with the expected product size at 946 bp. Another primer pair was specially designed for the last nucleotide (G) at the 3′ end of the forward primer (*cps2,14K*) to match with the SNP at position 483 of *cpsK* for identifying *S. suis* serotypes 2 and 14 with 209 bp amplicons (Figure 1). In combination with two primer pairs from a previous study [30], the new multiplex PCR was able to differentiate serotypes 1, 1/2, 2, 14 from each other.

The optimal conditions of the multiplex PCR assay were initially determined using genomic DNA of reference *S. suis* serotypes 1, 1/2, 2, and 14 as a template. The results showed that the optimal primer ratio was 5:0.5:0.5:2, with a final concentration of 1.25 µM for all *S. suis* specific primers, 0.125 µM for *cps1,14J* primers, 0.125 µM for *cps2,1/2J* primers, and 0.5 µM for *cps2,14K* primers, respectively. The annealing temperature was determined using PCR gradients ranging from 58–64 °C and showed the best optimal annealing temperature at 61 °C. 

**Figure 2:**
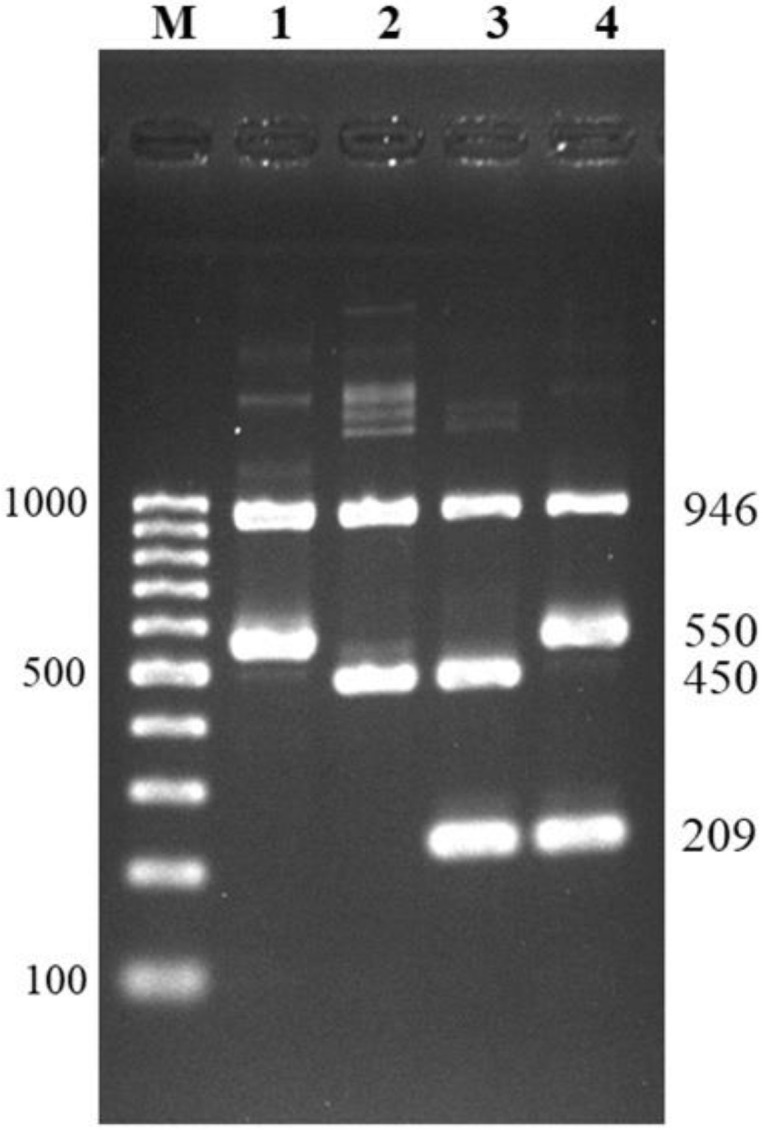
Multiplex PCR for differentiation of serotypes 1, 1/2, 2, and 14. Lane M = 100 bp DNA ladder; lane 1 = serotype 1; lane 2 = serotype 1/2; lane 3 = serotype 2; lane 4 = serotype 14.

The result interpretation for differentiation of serotype 1 from 14 and serotype 2 from 1/2 is shown in Figure 2. All serotypes could be identified as expected by gel electrophoresis: *S. suis* serotype 1 showed two positive bands at 946 bp of *recN* gene and 550 bp of *cps1,14J* gene; serotype 14 showed three positive bands at 946 bp of *recN* gene, 550 bp of *cps1,14J* gene, and 209 bp of *cps14K* gene; serotype 1/2, showed two positive bands at 946 bp of *recN* gene, and 450 bp of *cps2,1/2J* gene; and serotype 2 showed similar bands as serotype 1/2 with an additional positive band at 209 bp of *cps2K* gene. Using the developed method, serotypes 1, 1/2, 2, and 14 were easily discriminated within a single reaction of multiplex PCR.

### 2.2. Specificity and LOD of Multiplex PCR

**Figure 3:**
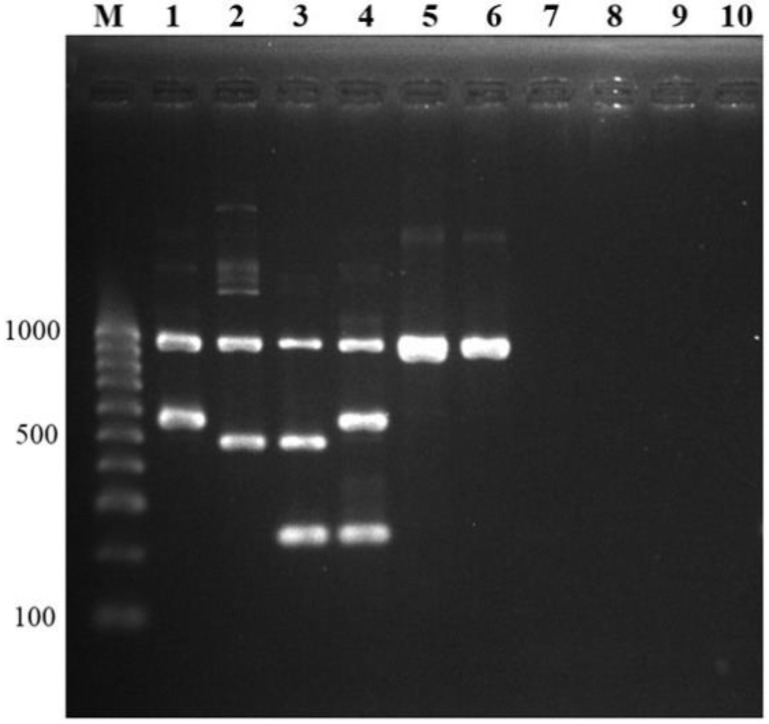
Specificity testing. Multiplex PCR was tested for cross-reactivity between other *S. suis* serotypes, *S. suis*-like strains (former *S. suis* serotypes), and other bacterial genera and species. Lane M = 100 bp DNA ladder; lane 1, 2, 3, 4 = positive controls (serotype 1, serotype 1/2, serotype 2, serotype 14); lane 5, 6 = other *S. suis* serotypes (serotype 15, serotype 19); lane 7, 8 = *S. suis*-like strains (former serotype 22, former serotype 32); lane 9, 10 = other bacterial genera (*Acinetobacter baumannii*, *Erysipelothrix rhusiopathiae*, respectively).

To determine the cross-reactivity to other *S. suis* serotypes of developed multiplex PCR, 29 serotypes of *S. suis* reference strains and *S. suis*-like strains; -serotypes 20, 22, 26, 32, and 34 were used. All 29 serotypes showed a positive band and amplified only the 946 bp of *recN* gene, whereas *S. suis*-like strains could not be amplified, as expected (Figure 3). The aforementioned serotypes 1, 1/2, 2, and 14 were also shown in the correct differentiation. In addition, no cross-reaction was observed with the other 48 bacterial genera and 6 *Candida* species tested. From these results, 100% specificity for *S. suis* identification and *S. suis* serotypes 1, 1/2, 2, and 14 differentiation of a new multiplex PCR were derived.

**Figure 4:**
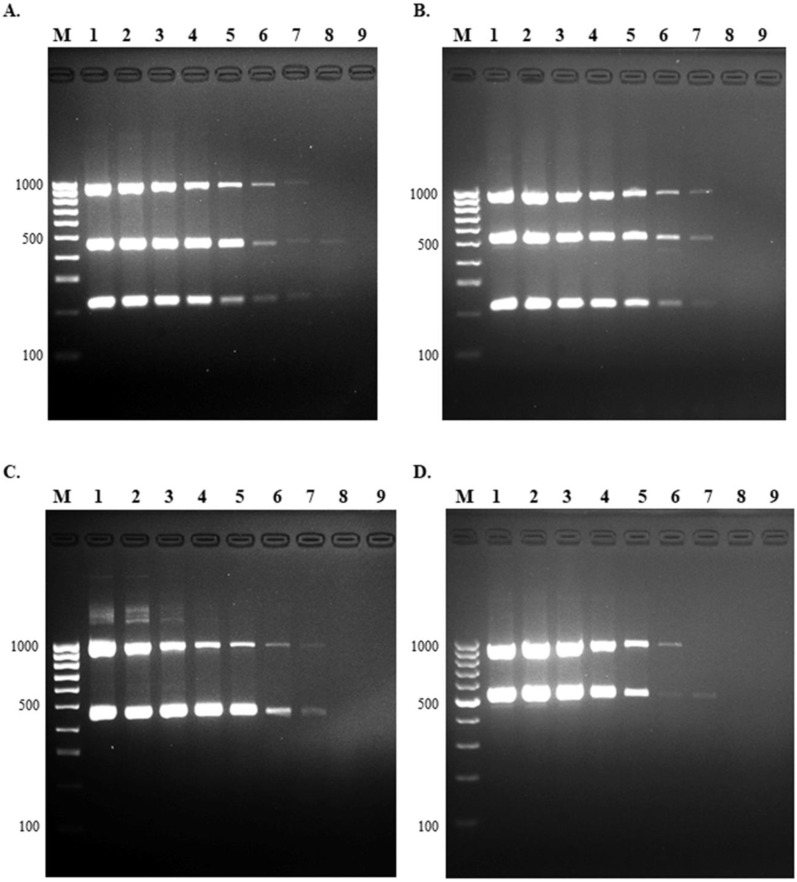
Limit of detection of multiplex PCR. The LOD was evaluated using 10-fold serial dilution of standard strains of *S. suis* (**A**) serotype 2, (**B**) serotype 14, (**C**) serotype 1/2, and (**D**) serotype 1. Lane M = 100 bp DNA ladder; lane 1 = 10^8^ CFU; lane 2 = 10^7^ CFU; lane 3 = 10^6^ CFU; lane 4 = 10^5^ CFU; lane 5 = 10^4^ CFU; lane 6 = 10^3^ CFU; lane 7 = 10^2^ CFU; lane 8 = 10^1^ CFU, and lane 9 = no template control.

The limit of detection for the multiplex PCR assay was performed using a 10-fold serial dilution of each serotype (1, 1/2, 2, and 14) starting at 10^8^ CFU/mL. The detection limit of this assay was 10^2^ CFU for serotypes 1/2, 2, 14, and 10^3^ CFU for serotype 1 (Figure 4).

### 2.3. Evaluation of Multiplex PCR

**Figure 5:**
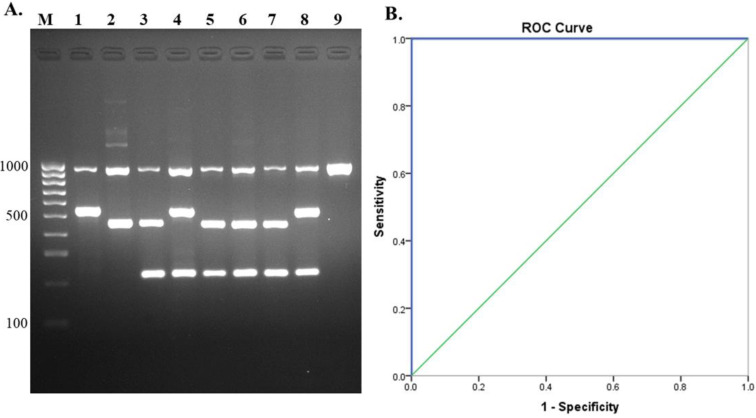
Evaluation of multiplex PCR using human and pig isolates. (**A**) Gel electrophoresis of isolates tested, lane M = 100 bp DNA ladder; lane 1, 2, 3, 4 = positive controls (serotype 1, serotype 1/2, serotype 2, serotype 14); lane 5 = isolate 1; lane 6 = isolate 2; lane 7 = isolate 3; lane 8 = isolate 4, and lane 9 = isolate 5. (**B**) Receiver operating characteristics (ROC) curve for evaluation of multiplex PCR with *S. suis* isolates.

A total of 190 *S. suis* isolates from humans and pigs were analyzed by the multiplex PCR assay. All of these isolates were also serotyped using PCR serotyping [31] combined with PCR-RFLP [34] and coagglutination serological testing [28]. Of the 190 isolates, our multiplex PCR identified serotype 2 in 126 isolates, 45 isolates as serotype 14, 2 isolates as serotype 1, and a total of 17 isolates of serotypes 3, 4, 5, 7, 9, 11, 15, 16, 18, 19, 24, 28, and 31 were identified as *S. suis* (Figure 5A). The results of the assays revealed 100% accordance with serological and PCR serotyping methods. Hence, diagnostic sensitivity was 100% (98.08–100%, 95% CI) and PPV was 100%, whereas specificity and NPV could not be calculated due to all tested isolates being *S. suis*. The ROC curve showed that the AUC = 1.0 (Figure 5B). The kappa value for agreement between our multiplex PCR and PCR serotyping, combined with PCR-RFLP and serological testing, was in almost perfect agreement, κ = 1.00, (0.81–1.00, 95% CI, *p* < 0.05). The developed assay showed high sensitivity and accuracy.

### 2.4. Direct Detection of S. suis from Clinical Samples

**Figure 6:**
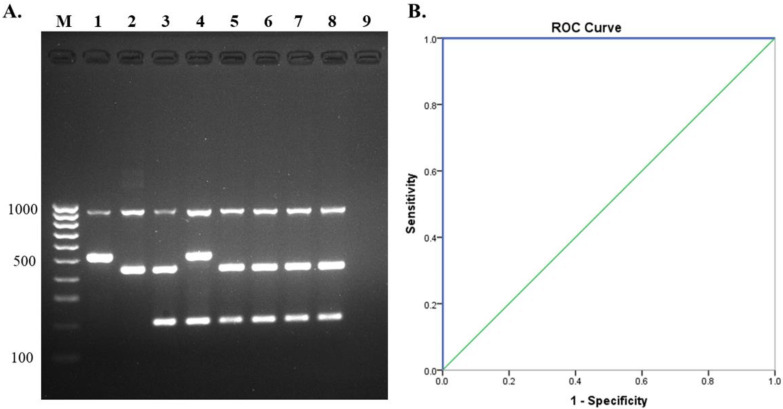
Direct detection of *S. suis* from human clinical specimens. (**A**) Evaluation of multiplex PCR using hemoculture Lane M = 100 bp DNA ladder, Lane 1, 2, 3, 4 = positive controls (serotype 1, serotype 1/2, serotype 2, serotype 14), Lane 5, 6, 7, 8 = hemoculture samples, and Lane 9 = no template control, (**B**) receiver operating characteristics (ROC) curve for direct detection of *S. suis* from clinical specimens.

A total of 125 patient hemocultures and one CSF sample from three hospitals in the Northern part of Thailand were included in this experiment. The multiplex PCR assay revealed that 65 hemocultures and one CSF were *S. suis* positive for serotype 2 (100%), while the other 60 hemocultures were *S. suis* negative (Figure 6A). Both *S. suis* positive and negative results were in 100% accordance with culture results. Furthermore, all serotype results from the multiplex PCR assay were in concordance with serology results. Therefore, sensitivity = 100% (94.56–100%, 95% CI), specificity = 100% (94.04–100%, 95% CI), PPV = 100%, and NPV = 100%, respectively. The ROC curve demonstrated that AUC was 1.0 (Figure 6B). The Kappa values for multiplex PCR and culture, PCR serotyping and PCR-RFLP, and serological testing were revealed as in almost perfect agreement, κ = 1.00, (0.81–1.00, 95% CI, *p* < 0.05). All these results revealed that the developed assay could be applied to direct detection of clinical specimens, especially from positive hemoculture and CSF, although further testing of more CSF samples is still needed to be performed.

## 3. Discussion

In this study, we developed a new multiplex PCR assay that detected true *S. suis* species along with the simultaneous differentiation of serotypes 1, 1/2, 2, and 14 from each other within a single reaction. Differentiation between serotypes 2 and 1/2, and serotypes 1 and 14, is challenging for PCR assays as these pairs of serotypes possess high similarity of the *cps* gene. The exact differentiation between serotypes 2 and 1/2, as well as between 1 and 14, is important for epidemiological investigation, monitoring dynamic serotype changes. Furthermore, serotypes 2 and 14 are profoundly associated with zoonotic disease [14]. 

*S. suis* serotypes are traditionally classified by coagglutination or agglutination tests using serotype-specific antisera. These techniques are simple; however, the production of specific antisera is time-consuming, expensive, laborious, and only available in reference laboratories. Cross-reaction also occurred in some serotypes; serotype 1/2 with serotypes 1 and 2, and serotypes 1 with serotype 14 [28]. Molecular PCR-based serotyping targeted the *cps* gene does not require expensive antisera, can be applied in many laboratories and is an alternative way for serotyping *S. suis*. However, these assays are not able to differentiate between serotype 2 from 1/2 and serotype 1 from 14.

Lately, it has been reported that a single nucleotide polymorphism (G to C/T) at position 483 of the *cpsK* gene is detectable using a short-read whole-genome sequencing and can be used to differentiate among these four serotypes [32]. The whole-genome sequencing approach is highly accurate; however, the cost per sample is high, time-consuming, and probably only available in some laboratories. Recently, the high-resolution melting curve assays, PCR-RFLP and MAMA-PCR, based on *cpsK,* were reported [33,34,35]. These methods could be rapid and sensitive, but a multi-reaction step is required to differentiate serotype 2 from 1/2 as well as 1 from 14. In addition, some techniques required a special instrument or another reagent, such as for the restriction of endonucleases.

Herein, we describe a simple multiplex PCR assay differentiating these four serotypes from each other based on the *cps1,14J*, *cps2,1/2J*, and *cps2,14K* genes within a single reaction. Furthermore, our assay included the primer targeting *recN* gene to specifically identify *S. suis*. The presence of two positive bands at the *recN* and *cps1,14J* genes for serotype 1, and the same for serotype 14 with an additional 209 bp of *cps14K* gene. Similarly, serotypes 1/2 and 2 are positive for the *recN* and *cps2,1/2J* genes with an additional 209 bp of the *cps2K* gene for serotype 2. The *cpsK* primer used in this study was highly specific for serotypes 2 and 14. Even with one nucleotide difference, it cannot amplify serotypes 1 and 1/2. Furthermore, a short product size enables rapid amplification and reduces turnaround time. Other *S. suis* serotypes, apart from these four, are positive for the *recN* gene, whereas *S. suis*-like strains are not amplified by our PCR. This is easy to perform; no additional steps, special instruments, or reagents are required. Our multiplex PCR assay also showed high sensitivity. No cross-reactions with the *S. suis*-like strains, other bacterial, or fungal species were observed. 

Traditional serotyping using the agglutination test is still considered a reference method. Most *S. suis* isolates from pigs are a loss of capsular expression after culture; in these cases, the agglutination test is unable to serotype [35]. However, multiplex PCR targeting of the *cps* gene can resolve to predict serotypes. For example, unencapsulated *S. suis* serotype 31 was not serotyped by coagglutination but could identify serotype 31 by using multiplex PCR [21]. Unencapsulated strains (due to mutations in the *cps* locus) have been mostly associated with endocarditis in pigs [36]. Furthermore, a complete deletion of the *cps* locus presented in non-typeable strains was recovered from diseased pigs [37]. Furthermore, a human case of *S. suis* infection caused by an unencapsulated strain has been found in Thailand, and a loss of capsular expression is due to a disrupted mutation in the *cpsE–cpsK* region [38]. In these cases, our multiplex PCR targeted to *cpsJ* and *cpsK* could be unable to serotype; however, including a species-specific *recN* gene can still identify *S. suis*, and a low possibility of a false-negative would be detected compared to only serotype-specific PCR assays. 

Multiplex PCR is a simple, rapid, low-cost method that can be applied in most laboratories. To the best of our knowledge, this is the first report of simultaneous single multiplex PCR differentiating *S. suis* serotypes 1, 1/2, 2, and 14. Our multiplex PCR assay combined with the previously published multiplex PCR assay [31] will be achieved for serotyping of all true *S. suis*. Furthermore, it can overcome the limitation of traditional serotyping that could only perform in reference laboratories and required the expensive specific antisera. Note that our PCR assay was limit with few isolates of the serotypes 1 and 1/2. Notably, more field isolates of these *S. suis* serotypes are required to further verify the assay. Since these two serotypes have a very low prevalence in pigs in Thailand (serotype 1 (1.5%), serotype 1/2 (0.5%)) [23], not many samples were included for evaluation of multiplex PCR assay. Collectively, human infections with these two serotypes have never been reported in Thailand, or the globe, based on the fact that serotype 1 and serotype 1/2 isolates have not yet been recovered or confirmed [4,26]. Although two human cases associated with suspected *S. suis* serotype 1 has been documented but was established using biochemical criteria only. These strains could not be confirmed as serotype 1 by the standard technique; therefore, it is still in question [1].

As *S. suis* infection mostly occurs asymptomatically in the early stages, rapid disease progression can lead to death within a short period, especially in splenectomized patients (patients died within 12 h [39], or 23 days [40], after hospital admission). Therefore, early and accurate diagnosis is critically important. The routine identification methods, including culture and biochemical tests, are time-consuming and sometimes showed controversy, resulting in underdiagnoses. Other alternative methods such as the Vitek II system, Phoenix System, and MALDI-TOF have been widely used for the identification of *S. suis*. However, these methods are performed from pure colonies and need a culture step. In addition, some systems probably misidentify *S. suis* as other *Streptococcal* species [41,42,43]. Due to these limitations, rapid and accurate diagnostic methods are needed.

The developed multiplex PCR assay enables us to identify and serotype *S. suis* directly from positive hemoculture and CSF with no culture step. Notably, hemoculture contain PCR inhibitors such as sodium polyanethol sulfonate (SPS), a common additive to automated hemoculture media that affects PCR reactions even when used with specific commercial extraction kits for blood [44]. Hence, extracted genomic DNA by a 1:10 dilution with sterile water could be useful to avoid PCR failure. Further testing of more CSF samples and other clinical specimen types such as synovial fluid, tissues, and pleural fluid are needed to verify the effectiveness of the multiplex PCR. Our multiplex PCR assay will be more convenient than the culture method owing to the 2–3 hour turnaround time. Unlike the culture method, which takes at least 2–3 days to get a result. However, gel electrophoresis is still needed, and other approaches will be needed to develop point-of-care testing (POCT) for *S. suis* detection in the future. 

## 4. Materials and Methods

### 4.1. Bacterial Strains and Growth Conditions

In order to determine the optimal conditions of multiplex PCR, reference strains of *S. suis* serotypes 1, 1/2, 2, and 14 were included. Reference strains of 29 *S. suis* serotypes and the former *S. suis* serotypes 20, 22, 26, 32, and 34 were used for specificity testing. Moreover, a total of 190 human (*n* = 165) and pig (*n* = 25) isolates, which have been previously serotyped using either antisera or PCR, were included in this study. We also included 48 species of other bacterial strains and 6 *Candida* species to observe possible cross-reactions (Table 1). *S. suis* was cultured onto 5% defibrinated sheep blood agar plates and incubated at 37 °C, 5% CO_2_ for 18–24 hours. Other bacterial species were grown overnight at 37 °C, 5% CO_2_, on 5% defibrinated sheep blood agar or chocolate agar, depending on their optimal medium. 

### 4.2. Genomic DNA Extraction

Genomic DNA of *S. suis* and other bacterial strains were extracted using a heat-lysis method with some modification [45]. Briefly, a loopful of the bacterial colonies were suspended into 30 µL lysis buffer (a mixture of 0.25% (vol/vol) sodium dodecyl sulphate (SDS), 0.05 M sodium hydroxide (NaOH) and distilled water), mixed by vortex and heated at 95 °C for 15 min, followed by centrifugation at 12,000× *g* for 5 min. Finally, the supernatant was transferred into a new tube, and we added 200 µL of sterile distilled water. The extracted genomic DNA was kept at −20 °C until used. 

Genomic DNA from hemoculture was extracted using a NucleoSpin^®^ Blood Mini kit (Macherey-Nagel, Düren, Germany) according to the manufacturer’s instructions. To remove PCR inhibitors, genomic DNA extracted from hemoculture were diluted to 1:10 with sterile distilled water prior to use as a template for PCR. Cerebrospinal fluid (CSF) was centrifuged, and the pellet was further processed to obtain genomic DNA using the heat-lysis method.

### 4.3. Multiplex PCR

In this study, two new primer pairs were designed to identify only *S. suis* species and discriminate between serotypes 1, 1/2, 2, and 14. To identify *S. suis* specifically, *recN* sequences of available *S. suis* serotypes and *S. suis*-liked strains were obtained from NCBI (https://www.ncbi.nlm.nih.gov/, accessed on 11 June 2020) and aligned with the Clustal Omega program (https://www.ebi.ac.uk/Tools/msa/clustalo/, accessed on 11 June 2020). The primer binding sites were analyzed and designed. Discrimination between four serotypes and a new pair of primers were designed to target the *cps2K* and *cps14K* genes that possess point mutations at position 483 (G nucleotide). This primer pair was designed using the Primer 3 PCR primer designing tool (https://primer3.ut.ee/, accessed on 11 June 2020). Two pairs of primers (*cps1,14J*, and *cps2,1/2J*) from previous studies were also included in the reaction [30]. The primer sequences used in this study are shown in Table 2.

Optimal conditions of the multiplex PCR, such as annealing temperature, primer concentration, and primer ratio, were determined using genomic DNA of reference *S*. *suis* serotypes 1, 1/2, 2, and 14 as a template. The PCR mixture contained 1× Quick Taq^®^ HS Dye Mix (Toyobo Life Sciences, Osaka, Japan), 1.25 µM for all *S. suis* specific primers, 0.125 µM for *cps1,14J* primers, 0.125 µM for *cps2,1/2J* primers, 0.5 µM for *cps2,14K* primers and 1 µL (100 ng) of template DNA in a total of 20 µL per reaction. The thermal profile of PCR was used based on the optimal condition of multiplex PCR: initial activation of DNA polymerase at 95 °C for 3 min, 35 cycles of denaturation at 95 °C for 20 s, primer annealing and extension at 61 °C for 90 s, and final extension at 72 °C for 5 min. The PCR products were analyzed by agarose gel electrophoresis (2% agarose in 0.5× Tris-Acetate-EDTA (TAE) buffer) for 40 min. The gels were pre-stained with RedSafe™ Nucleic Acid Staining Solution (iNtRON Biotechnology, Inc, Seongnam-Si, Korea) and visualized under UV light (G: BOX F3, SYNGENE, Cambridge, England). The sizes of PCR products were determined by comparison with a molecular size standard (GeneRuler 100 bp DNA ladder, Thermo Fisher Scientific, Waltham, MA, USA). 

### 4.4. Limit of Detection (LOD) 

The limit of detection of multiplex PCR was determined using a 10-fold serial dilution of each *S. suis* serotype (serotypes 1, 1/2, 2, and 14). Briefly, the overnight cultures of four *S. suis* serotypes were harvested and washed thrice with 1× phosphate-buffered saline (PBS). Then, the pellets were suspended with 1× PBS and adjusted to the original concentration of 10^8^ CFU/mL by measuring turbidity at an optical density of 600 nm (OD_600_). Measuring OD_600_ at 0.3 for serotype 2, 0.1 for serotype 14, 0.5 for serotype 1/2, and 0.7 for serotype 1 were performed. Starting from 10^8^ CFU/mL, 10-fold serial dilutions were made until reaching 10 CFU/mL. Genomic DNA was extracted from 1 mL of each dilution using the heat-lysis method. At the same time, each dilution was also plated on blood agar and incubated at 37 °C, 5% CO_2_ overnight for the colony count, in order to determine the minimum colony-forming unit required for multiplex PCR.

### 4.5. Evaluation of Multiplex PCR Using S. suis Colonies from Human and Pig Isolates and Direct Detection of S. suis from Clinical Specimens

The multiplex PCR was validated using a pure culture of a total of 190 *S. suis* samples isolated from human clinical specimens and healthy pig tonsils (which have been previously serotyped by PCR serotyping [31]) combined with the PCR-RFLP method [34] and coagglutination serological testing [28]. Among 190 culture isolates, 126 isolates of serotype 2, 45 isolates of serotype 14, 2 isolates of serotype 1, and a total of 17 isolates of serotypes 3, 4, 5, 7, 9, 11, 15, 16, 18, 19, 24, 28, and 31 were included.

Furthermore, a total of 125 hemoculture samples and one CSF sample were also included for direct detection of the assay. From culture and biochemical testing, 65 samples were identified as *S. suis*, and 60 samples were *S. suis*-negative (identified as other bacterial genera and species (*n* = 50), or no growth (*n* = 10)). One CSF sample was also identified as *S. suis*. Diagnosis accuracy was measured in terms of sensitivity, specificity, positive predictive value, and negative predictive value, compared to either culture and biochemical testing or serological testing and PCR serotyping. The receiver operating characteristic curve (ROC) was performed. In order to describe the agreement between new multiplex PCR and culture or serotyping methods, Cohen’s kappa coefficient with a 95% confidence interval (95% CI) was calculated. Kappa values were interpreted as follow: 0.00–0.20 as poor agreement; 0.21–0.40, fair agreement; 0.41–0.60, moderate agreement; 0.61–0.80, good agreement; 0.81–1.00 almost perfect agreement [46]. All statistical analyses were performed using the SPSS statistical package, release 22.0 (SPSS Inc., Chicago, IL, USA).

## 5. Conclusions

The multiplex PCR assay in the current study provides an easy, rapid, cost-effective, and high accuracy method to identify and differentiate *S. suis* serotypes 1, 1/2, 2, and 14 from each other within a single reaction. Direct detection from hemoculture and CSF also revealed high sensitivity, specificity, PPV, NPV, and accuracy, with almost perfect agreement (κ = 1.0), compared to culture and serotyping results. This facilitates a rapid diagnosis and can be applied for an effective treatment, investigation, and control of *S. suis* infection in both humans and pigs.

## Figures and Tables

**Table 1:** Other bacterial and fungal species used in specificity testing.

Microorganisms	Species
Gram-positive bacteria (n = 22)	*Streptococcus pneumoniae* ATCC 49619, *Streptococcus mutans* ATCC 18777, *Streptococcus agalactiae* ATCC 17129, *Streptococcus pyogenes* ATCC 19615, *Streptococcus bovis* (*n* = 2), *Streptococcus oralis*, *Streptococcus mitis*, *Staphylococcus aureus* ATCC 25923, *Staphylococcus epidermidis* ATCC 14990, *Staphylococcus saprophyticus* ATCC 15305, *Staphylococcus haemolyticus* ATCC 29970, *Staphylococcus intermedius* group (*n* = 3), *Micrococcus* spp., *Enterococcus faecalis* ATCC 4736, *Enterococcus faecium* ATCC 4743, *Bacillus cereus*, *Listeria monocytogenes*, *Erysipelothrix rhusiopathiae*, *Corynebacterium* spp.
Gram-negative bacteria (*n* = 26)	*Escherichia coli* ATCC 25922, *Pseudomonas aeruginosa* ATCC 27853, *Klebsiella pneumoniae* ATCC 700603, *Klebsiella oxytoca*, *Proteus mirabilis*, *Proteus vulgaris*, *Proteus peneri*, *Burkholderia cepacia*, *Acinetobacter baumannii* ATCC 19606, *Acinetobacter lwoffii*, *Pasteurella multocida*, *Enterobacter aerogenes*, *Enterobacter cloacae*, *Salmonella* Choleraesuis, *Salmonella* Enteritidis, *Salmonella* Typhi, *Salmonella* Paratyphi, *Stenotrophomonas maltophilia*, *Haemophilus influenzae* ATCC 49247, *Neisseria meningitidis*, *Achromobacter xylosoxidans*, *Aeromonas hydrophilia*, *Shigella flexneri*, *Shigella boydii*, *Shigella sonnei*, *Haemophilus parainfluenzae*
Fungal *Candida* spp. (*n* = 6)	*Candida albicans* ATCC 90028, *Candida tropicalis*, *Candida glabrata*, *Candida parapsilosis* ATCC 22019, *Candida guilliermondii*, *Candida krusei*

**Table 2:** Primers and target genes used in the multiplex PCR assay.

Primer Name	Sequence (5′–3′)	Target Gene	PCR Product Size	Result	Reference
*cps1,14J*	F: AATCATGGAATAAAGCGGAGTACAG R: ACAATTGATACGTCAAAATCCTCACC	*cps1J, cps14J*	550	Serotypes 1 and 14	[30]
*cps2,1/2J*	F: GATTTGTCGGGAGGGTTACTTG R: TAAATAATATGCCACTGTAGCGTCTC	*cps2J, cps1/2J*	450	Serotypes 2 and 1/2	[30]
*cps2,14K*	F: CTTTGTGGTGGCCTGG R: AATGGAAGCGATGGTCAG	*cps2K, cps14K*	209	Serotypes 2 and 14	This study
All *S. suis* specific	F: TCCTTTGAAAATAGCAGAGCTC R: GCGGATAATATCTTCTAAAACA	*recN*	946	*S. suis*	This study

F, forward; R, reverse.

## Data Availability

Not applicable.
